# Three-component NiO/Fe_3_O_4_/rGO nanostructure as an electrode material towards supercapacitor and alcohol electrooxidation

**DOI:** 10.1016/j.heliyon.2024.e39399

**Published:** 2024-10-15

**Authors:** Mohammad Bagher Askari, Mohammad Taghi Tourchi Moghadam, Parisa Salarizadeh

**Affiliations:** a Department of Semiconductor, Institute of Science and High Technology and Environmental Sciences, Graduate University of Advanced Technology, Kerman, Iran; b Faculty of Electronics, Telecommunications and Informatics, and Advanced Materials Centre, Gdansk University of Technology, Ul. Narutowicza 11/12, 80-223, Gdansk, Poland; c High-Temperature Fuel Cell Research Department, Vali-e-Asr University of Rafsanjan, Rafsanjan, 7718897111, Iran

**Keywords:** NiO/Fe_3_O_4_/rGO, Supercapacitors, Alcohol fuel cells

## Abstract

A nanocomposite made of nickel oxide and iron oxide (NiO/Fe_3_O_4_) and its hybrid with reduced graphene oxide (rGO) as a conductive substrate with a highly functional surface (NiO/Fe_3_O_4_/rGO) was synthesized using a simple hydrothermal approach. This study addresses the challenge of developing efficient materials for energy storage and alcohol fuel cells. After confirming the synthesis through structural analysis, the potential of these nanocomposites as supercapacitor electrodes and catalysts for methanol and ethanol oxidation in alcohol fuel cells were evaluated. The synergy of combining the two metal oxides and adding rGO to the composite structure led to excellent electrocatalytic activity in alcohol oxidation. For the modified NiO/Fe_3_O_4_/rGO electrode in the methanol oxidation reaction (MOR), a current density of 450 mA/cm^2^ at 0.67 V and excellent catalyst stability of 98.7 % over 20 h in chronoamperometric analysis were observed. In the ethanol oxidation reaction (EOR), an oxidative current of 235 mA/cm^2^ at a peak potential of 0.76 V was seen, with catalyst stability of 96.4 % after 20 h. As a supercapacitor electrode, the NiO/Fe_3_O_4_ composite demonstrated a specific capacitance of 946 F/g, while NiO/Fe_3_O_4_/rGO showed 1155 F/g. The stability of these electrodes after 10000 GCD cycles was 83.6 % and 90.6 %, respectively. These findings suggest that the proposed structures are cost-effective and reliable alternatives for energy storage and production, suitable for alcohol fuel cells and supercapacitors.

## 1 Introduction

Addressing the energy crisis from fossil fuel depletion and pollution is critical. The shift toward clean fuels and renewable resources is essential to mitigate greenhouse gas emissions [1,2]. In energy storage, there's a growing demand for portable, high-efficiency devices like supercapacitors and fuel cells [3], which have become focal points in the search for sustainable solutions. Supercapacitors and fuel cells are among the high-efficiency and portable devices that have garnered Substantial focus.

Fuel cells transform chemical energy into electrical output directly, while supercapacitors can store much more energy than regular capacitors. The efficiency of these devices heavily depends on the materials of their electrodes. Supercapacitors and alcohol fuel cells have been extensively studied as promising technologies to overcome the energy crisis in recent years [4,5]. Supercapacitors consist of two porous electrodes, an ion-conducting membrane, and an electrolyte [6,7]. Energy storage is enhanced by the electric field between the electrodes and electrolyte, with capacity influenced by the electrode material, porosity, and morphology [5]. Supercapacitors offer higher energy density than solid-state and conventional capacitors and greater power density than batteries, effectively bridging the gap between them. Their rapid charge and discharge times are key advantages [8]. Based on their energy storage mechanisms, supercapacitors are divided into two categories: Electrical Double-Layer Capacitors (EDLCs) and pseudocapacitors [9,10]. The storage mechanism of EDLCs involves electrostatic charge adsorption on the electrode surface, with no charge transfer or Faradaic reactions occurring between the electrode and electrolyte [11,12]. In this configuration, two porous electrodes are submerged in a conductive electrolyte and are separated by a mechanical separator [13,14]. Carbon-based structures are the most famous electrode materials with this mechanism [15]. Unlike EDLCs, pseudocapacitors store electrical energy through reversible redox reactions occurring on the electrode surfaces [16,17]. The electron transfer in pseudocapacitors, driven by a detached and adsorbed ion [12], does not result in chemical bonding between the ion and the electrode atoms [14]. Pseudocapacitors, with their Faradaic current, can achieve higher energy density compared to EDLCs [18]. The faster Faradaic reactions in pseudocapacitors, as opposed to batteries, are attributed to the lack of chemical interaction between the adsorbed ion and electrode atoms. Transition metal oxides and conductive polymers typically exhibit this mechanism [14,19].

Using metal oxides as catalysts for alcohol oxidation and as cost-effective, high-efficiency electrode materials with excellent stability in supercapacitor structures is of great interest [20,21]. Numerous studies indicate that these materials are theoretically highly efficient in the energy science field, and recent experimental studies confirm this theory [[22], [23], [24], [25]]. We are witnessing numerous studies in this field, attracting many researchers to this vast and fascinating science.

Although metal oxides are generally inexpensive and abundant, and can be synthesized in the laboratory using simple and efficient methods, they also have drawbacks when used as catalysts and electrode materials [[26], [27], [28]]. A suitable composite for use in fuel cell and supercapacitor structures must have good electrical conductivity and a suitable active surface [29]. Metal oxides do not have very good electrical conductivity, and another drawback is their very low stability in acidic environments. Additionally, processing to create electrodes from these materials is challenging [30,31].

To minimize these shortcomings, there are two approaches: 1) combining of these materials with substances that have good electrical conductivity, such as conductive polymers or certain carbon structures; 2) combining these metal oxides as binary or multi-component structures due to creating synergistic effect. In the present study, both approaches were considered. A three-component composite consisting of iron and nickel metal oxides was synthesized and the electrical conductivity and efficient area of the metal oxide-based composite was enhanced by hybridizing them with rGO.

Iron oxide, as a magnetic metal oxide is frequently used and is among the most prevalent materials. in the field of catalysts and has always attracted researchers' attention [[32], [33], [34]]. The inherent catalytic properties of iron have been proven in many studies, and its extensive applications in various fields of biology, engineering sciences, energy, and electrochemistry have been investigated [[35], [36], [37], [38], [39], [40]]. The use of iron oxide into the framework of high-efficiency catalysts for various fuel cells [41,42], in the structure of supercapacitor electrodes [43], and Different electrochemical energy storage systems [44] are among these studies.

In this study, the NiO/Fe_3_O_4_/rGO composition was chosen due to the complementary properties of its components. Nickel oxide (NiO) provides excellent electrocatalytic activity, while iron oxide (Fe_3_O_4_) enhances electron transfer during electrochemical reactions [45]. However, both oxides have limitations in electrical conductivity, which are addressed by incorporating reduced graphene oxide (rGO). rGO significantly improves the composite's conductivity and active surface area, resulting in a material that is highly efficient, stable, and cost-effective for applications in supercapacitors and alcohol fuel cells [[46], [47], [48], [49]].

## 2 Experimental

### 2.1 Materials and equipment

All precursors, including iron sulfate, butanol, nickel chloride, trisodium citrate, urea, potassium hydroxide, thiourea, methanol, and ethanol with a purity of over 99 %, were purchased from Merck. A 5 % Nafion solution was obtained from Sigma-Aldrich. X-ray diffraction analysis was performed using X'Pert PRO Powder Diffractometer (PANalytical) and Raman spectroscopy analysis was conducted with Raman Takram P50C0R10. Scanning electron microscopy (SEM) images and EDX mapping were done using SEM TESCAN, MIRA4, and electrochemical analyses were carried out with a Metrohm Potentiostat/Galvanostat model AUTOLAB PGSTAT-302N.

### 2.2 Synthesis of NiO/Fe_3_O4 and NiO/Fe_3_O_4_/rgo nanocatalysts via hydrothermal method

To synthesize NiO/Fe_3_O_4_, 0.28 g of FeSO_4_·7H_2_O was dissolved in a solution containing 30 mL of deionized water, 20 mL of ethanol, and 2 mL of butanol, stirring for 30 min to obtain a clear solution. Then, 0.5 g of NiCl_2_·6H_2_O, 0.2 g of trisodium citrate (Na_3_C_6_H_5_O_7_), and 0.3 g of urea were added to the solution. Stirring was continued with a magnetic stirrer for another 30 min. The resulting mixture was placed into a 100 mL stainless-steel autoclave lined with Teflon and heated in an oven at 170 °C for 6 h. After cooling the reactor to 25 °C, the product was subjected to several washes with water and ethanol using centrifugation at 6000 rpm, then, it was dried in a vacuum oven at 60 °C for 4 h. Finally, the obtained powder was calcined at 400 °C for 3 h, resulting in the NiO/Fe_3_O_4_ nanocomposite.

To synthesize NiO/Fe_3_O_4_/rGO, the same procedure as for NiO/Fe_3_O_4_ was followed, but 0.05 g of GO was added in the first step along with 0.28 g of FeSO_4_·7H_2_O to the solution containing deionized water, ethanol, and butanol. The rest of the steps are identical to the NiO/Fe_3_O_4_ synthesis. The amount of GO used in the NiO/Fe_3_O_4_/rGO structure has been optimized, and the electrochemical results of the optimized sample are reported.

### 2.3 Preparation of the electrode

A 2 cm × 1 cm nickel foam was used as the working electrode. In the three-electrode system, a platinum wire with a diameter of 0.5 mm served as the auxiliary electrode, and an Ag/AgCl electrode acted as the reference electrode. A certain amount of NiO/Fe_3_O_4_ and NiO/Fe_3_O_4_/rGO were dispersed in a solvent containing Nafion (5 % solution), isopropyl alcohol, and deionized water by ultrasonication for 20 min. The resulting slurry, with a volume of 5 μL, was drop-cast onto the surface of the nickel foam and subsequently dried in a vacuum oven at 40 °C for 20 min.

## 3 Results and discussion

### 3.1 Characterization of NiO/Fe_3_O_4_ and NiO/Fe_3_O_4_/rGO nanocomposites

**Fig. 1:**
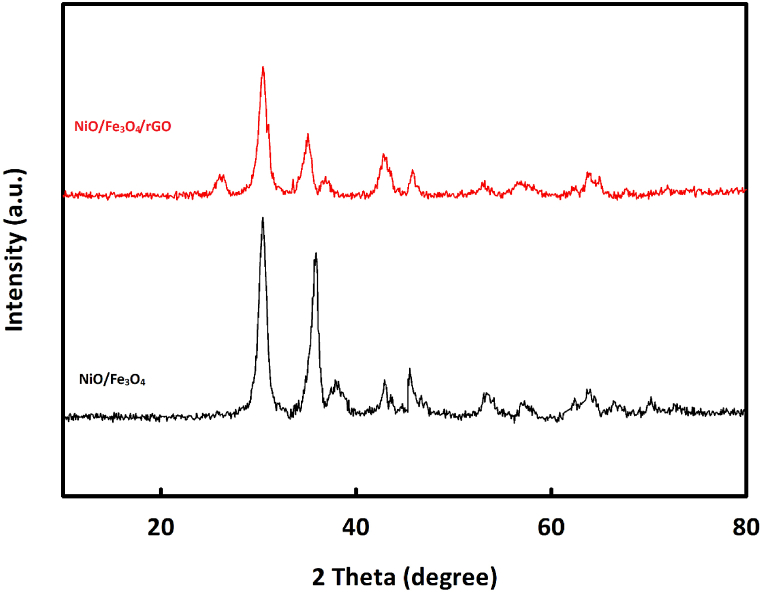
XRD patterns of NiO/Fe_3_O_4_ and NiO/Fe_3_O_4_/rGO.

The crystalline structure of the NiO/Fe_3_O_4_ and NiO/Fe_3_O_4_/rGO nanocomposites was evaluated using X-ray diffraction analysis (Fig. 1). In the X-ray diffraction pattern of the NiO/Fe_3_O_4_ nanocomposite, characteristic peaks of NiO and Fe_3_O_4_ are observed. The Fe_3_O_4_ diffraction pattern shows six characteristic peaks at diffraction angles of 30.4°, 35.8°, 42.9°, 53.3°, 56.6°, and 62.3°, corresponding to the Miller indices (220), (311), (400), (422), (511), and (044), respectively. This diffraction pattern matches perfectly with JCPDS No. 16–0619 [50]. NiO also shows three Recognizable peaks in the diffraction pattern at angles of 37.7°, 44.9°, and 63.7°, corresponding to Miller indices (111), (200), and (220). The crystalline structure of NiO is in perfect accordance with JCPDS No. 47–1049 [50]. In the X-ray diffraction pattern of the NiO/Fe_3_O_4_/rGO composite, in addition to the characteristic peaks of NiO and Fe_3_O_4_, a broad peak at about 26° belonging to rGO is also observed.

**Fig. 2:**
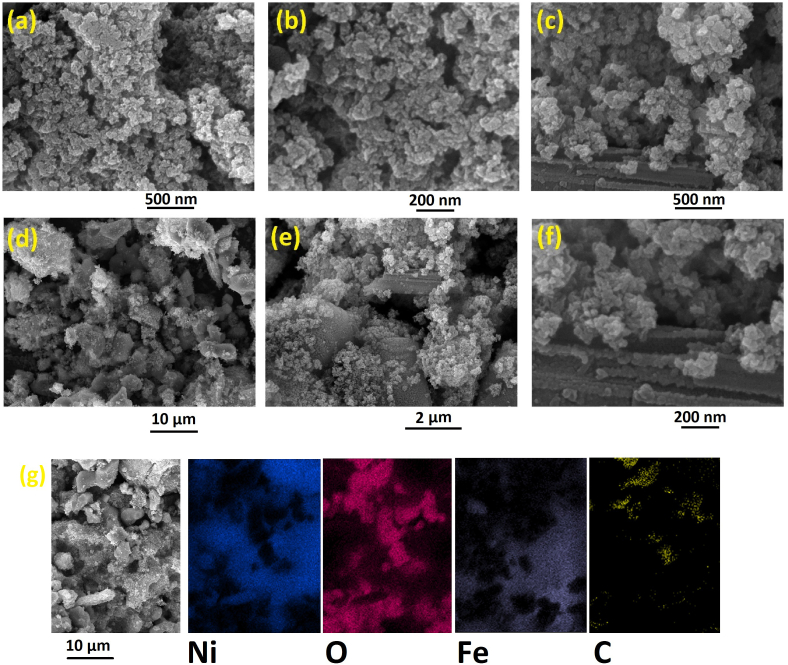
SEM images of NiO/Fe_3_O_4_ (a, b), NiO/Fe_3_O_4_/rGO (c–f) and EDX mapping analysis of NiO/Fe_3_O_4_/rGO (g).

The surface structure of the synthesized nanocomposites was examined by providing SEM images. In Fig. 2a–b, the NiO/Fe_3_O_4_ nanocomposite is shown, where nickel oxide and iron oxide are labeled with red lines. Structural porosity and the presence of cracks in the composite structure play a crucial role in the oxidation processes of methanol and ethanol and the penetration of KOH as the electrolyte. These pathways act as shortcuts, allowing the electrolyte and fuel to penetrate the composite's depth more quickly, thus exposing more of the composite's surface to the electrolyte. This results in greater stability of the composites in electrochemical processes and accelerates the release of activation energy of the catalyst.

In Fig. 2c–f, the composite consisting of NiO/Fe_3_O_4_ and rGO nanosheets is shown. These images clearly show that the metal oxides are uniformly distributed on the rGO surface. rGO, as a conductive substrate with a very good surface reactivity, facilitates electrochemical processes in energy production and storage.

The homogeneous spread of metal oxides on the rGO surface was also studied using EDX mapping analysis. In Fig. 2g, this uniform distribution of elements can be seen. Additionally, the existence of iron, nickel, oxygen, and carbon elements in the composite structure is confirmed by the results of this analysis.

**Fig. 3:**
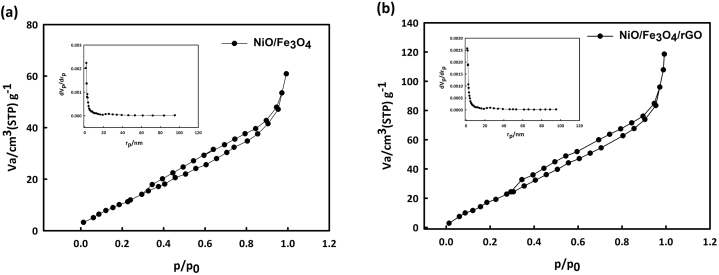
N_2_ adsorption-desorption isotherms and BJH pore size distribution of NiO/Fe_3_O_4_ (a) and NiO/Fe_3_O_4_/rGO (b)**.**

Using BET surface area analysis and BJH pore size, the porosity and pore size of the synthesized composites were investigated. Fig. 3(a, b) shows the nitrogen gas absorption-desorption diagrams and the distribution of pore size for NiO/Fe_3_O_4_ and NiO/Fe_3_O_4_/rGO nanostructures, respectively. Based on this analysis, the specific surface area for NiO/Fe_3_O_4_ and NiO/Fe_3_O_4_/rGO obtained 45.361 and 87.031 m^2^ g^−1^, respectively. Also, the mean pore size for the NiO/Fe_3_O_4_ and NiO/Fe_3_O_4_/rGO obtained at about 7.3, and 6.8 nm, respectively, which confirms that the pores are mesoporous.

### 3.2 Electrochemical studies

#### 3.2.1 Evaluation of NiO/Fe_3_O_4_/rGO composite as electrode for supercapacitors

**Fig. 4:**
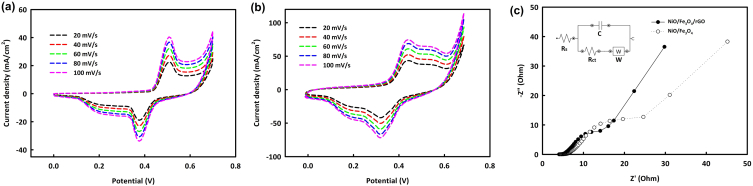
Cyclic voltammetry analysis of NiO/Fe_3_O_4_ (a), NiO/Fe_3_O_4_/rGO (b) in the potential range of 0–0.7 V at different scan rates (20–100 mV/s) and electrochemical impedance spectroscopy of NiO/Fe_3_O_4_ and NiO/Fe_3_O_4_/rGO (c).

The capability of NiO/Fe_3_O_4_ and NiO/Fe_3_O_4_/rGO nanocomposites as supercapacitor electrodes was evaluated in an alkaline medium (1 M KOH as the electrolyte). Cyclic voltammetry (CV) analysis was performed on the prepared electrodes across a potential range of 0–0.7 V with varying scan rates (20–100 mV/s). Fig. 4a and b correspond to this analysis for NiO/Fe_3_O_4_ and NiO/Fe_3_O_4_/rGO, respectively. As evident, the faradaic and capacitive currents increase with the scan rate. The electrochemical impedance spectroscopy (EIS) analysis is shown in Fig. 4c. The fitted equilibrium circuit is in inset of Fig. 4c. The equilibrium circuit contains charge transfer resistance (Rct), solution resistance (Rs), double layer capacitance (C), and Warburg impedance (W). The value of Rct for NiO/Fe_3_O_4_/rGO was observed to be 21 *Ω*, whereas for NiO/Fe_3_O_4_, R_ct_ was 33 *Ω*. The solution resistance was also recorded to be 3.8 *Ω*. Lower Rct of NiO/Fe_3_O_4_/rGO is due to the presence of rGO in the catalyst structure.

**Fig. 5:**
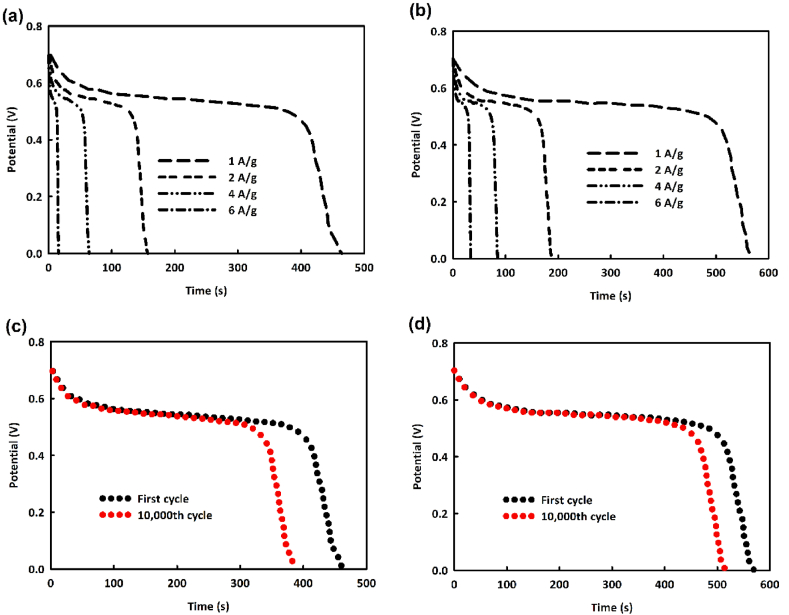
Galvanostatic charge-discharge analysis of NiO/Fe_3_O_4_ (a), NiO/Fe_3_O_4_/rGO (b) in the current densities of 1, 2, 4, and 6 A/g and 10,000 consecutive GCD of NiO/Fe_3_O_4_ (c) and NiO/Fe_3_O_4_/rGO (d).

In energy storage studies and evaluating electrode capability for supercapacitors, the GCD (galvanostatic charge-discharge) analysis is one of the most important and accurate analyses. Fig. 5a and b shows the GCD analysis for NiO/Fe_3_O_4_ and NiO/Fe_3_O_4_/rGO, respectively. This analysis was conducted at current densities of 1, 2, 4, and 6 A/g within a potential range of 0–0.7 V. As shown in the figures, the discharge time for NiO/Fe_3_O_4_/rGO at 1 A/g is 566 s, which is about 102 s longer than that for NiO/Fe_3_O_4_. The specific capacitance calculated from the GCD analysis for NiO/Fe_3_O_4_ and NiO/Fe_3_O_4_/rGO at this current density, using the equation $C=\frac{I\Delta t}{m\Delta V}$ [51] were 946 F/g and 1155 F/g, respectively.

To assess the stability of the NiO/Fe_3_O_4_ and NiO/Fe_3_O_4_/rGO electrodes, 5000 consecutive GCD cycles were performed (Fig. 5c and d). The stability of NiO/Fe_3_O_4_ was 83.6 %, and for NiO/Fe_3_O_4_/rGO, it was 90.6 %, which are considered good value. As seen in Fig. 5c, in the initial cycles, there is a greater drop in current density. Then, with the electrode surface's and interface's activation, this decreasing trend continues with a milder slope and eventually stabilizes. The electrode with rGO in its structure, due to its higher active surface area available to the electrolyte, more quickly provides its active sites to the electrolyte and reaches stability. Table 1 compares the performance of NiO/Fe_3_O_4_/rGO as a supercapacitor electrode with similar works. The results show that NiO/Fe_3_O_4_/rGO is comparable with other reported in terms of specific capacitance and stability.

**Table 1:** Comparison of the performance of NiO/Fe_3_O_4_/rGO with similar articles.

Electrode material	Supporting material	Electrolyte	C [F g^−1^]	Stability-cycle number	Ref.
**NiO/Fe**_**3**_**O**_**4**_**/rGO**	Ni foam	1M KOH	1155	90.6%-10000 GCD	This work
**NC@FO**	Ni foam	3 M KOH	1210	83.32 %- 10000 GCD	[52]
**Fe**_**2**_**N@Fe**_**3**_**O**_4_**NPs/NrGO**	Ni foam	2 M KOH	341.3	91.2 %- 10000 GCD	[53]
**Fe**_**3**_**O**_**4**_**–Bi**_**2**_**O**_**3**_	Ni foam	1 M KOH	1200	91 % - 7500 GCD	[54]
**Fe**_**3**_**O**_**4**_**/MWCNTs**	Graphite sheet	6 M KOH	492.3	165.7 % −1000 GCD	[55]
**NiSRu@NiO**	Ni foam	2 M KOH	1000	98%- 3000 GCD	[56]
**NiO/ZnO/GO**	Graphite paper	3 M KOH	1800	108%-1000 GCD	[57]
**Cu**_**2**_**O@MnO**_**2**_**@NiO**	FTO glass	5 M KOH	1092	95.24 % - 10000 GCD	[58]
**NiO@Mn**_**3**_**O**_**4**_**/rGO**	Ni foam	1 M KOH	533.97	75%- 2000 GCD	[59]
**P-Ni**_**2**_**MnO**_**4−x**_**@rGO**	Ni foam	2 M KOH	1344.7	94.9%- 10000 GCD	[60]

#### 3.2.2 Electrocatalytic activity of nanocatalysts in the methanol oxidation process

**Fig. 6:**
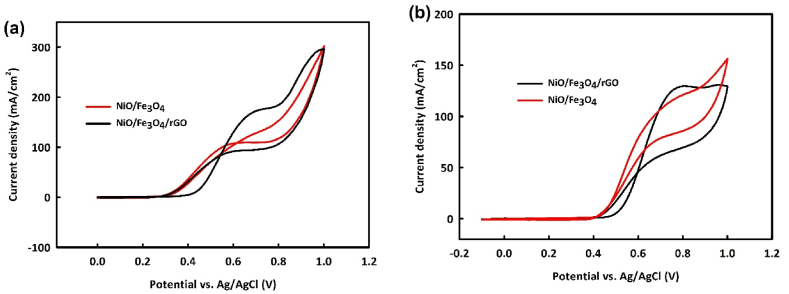
Cyclic voltammetry analysis of NiO/Fe_3_O_4_ (a) and NiO/Fe_3_O_4_/rGO (b) in the potential range of 0–1 V at the scan rates 20 mV/s in 0.2 M of Methanol/1 M KOH.

Metal oxides generally exhibit better catalytic activity for alcohol oxidation in alkaline media. To investigate the electrocatalytic activity of NiO/Fe_3_O_4_ and NiO/Fe_3_O_4_/rGO, 0.2 M methanol was added to a 1 M KOH solution. Fig. 6a and b shows the behavior of NiO/Fe_3_O_4_ and NiO/Fe_3_O_4_/rGO in the methanol oxidation reaction (MOR) and ethanol oxidation reaction (EOR), respectively. As shown in both figures, the catalysts exhibit relatively good electrocatalytic activity at this particular concentration, as evidenced by the alcohol oxidation peaks.

**Fig. 7:**
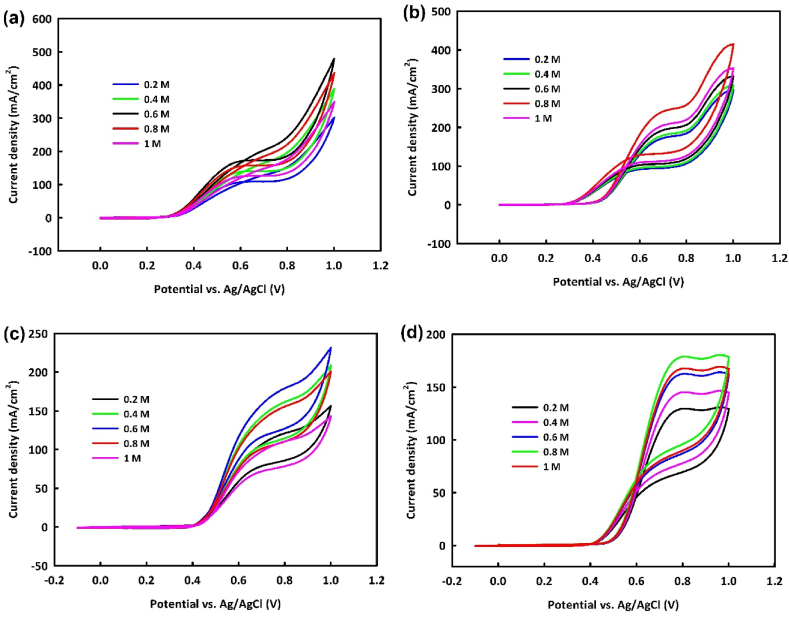
Cyclic voltammetry analysis in the potential range of 0–1 V at the scan rate of 20 mV/s in different concentrations of methanol for NiO/Fe_3_O_4_ (a) and NiO/Fe_3_O_4_/rGO (b) and ethanol for NiO/Fe_3_O_4_ (c) and NiO/Fe_3_O_4_/rGO (d) at the presence of 1 M KOH.

Due to the suitable catalytic activity of these catalysts, the optimization of the concentration of each alcohol will be addressed, and a proposed mechanism for methanol and ethanol oxidation will be presented. To examine the catalytic activity of NiO/Fe_3_O_4_ at various methanol concentrations, CV analysis was performed with the modified electrode at concentrations of 0.2–1 M methanol in a 1 M KOH alkaline medium, within a potential range of 1V and at a scan rate of 20 mV/s. As observed, with an increase in methanol concentration up to 0.6 M, the methanol oxidation peak current density increases. However, at 0.8 M and 1 M methanol, a decrease in current density is noted, likely due to catalyst surface saturation with increased methanol concentration (Fig. 7a). The oxidation peak current density for the NiO/Fe_3_O_4_/rGO modified electrode also shows an increasing trend up to 0.8 M methanol concentration, with a decrease observed at 1 M methanol. This may be attributed to the increased electrochemical active surface area owing to the inclusion of rGO in NiO/Fe_3_O_4_/rGO, leading to catalyst surface saturation at higher methanol concentrations (Fig. 7b).

In studying the behavior of synthesized catalysts in the EOR at concentrations of 0.2–1 M ethanol, NiO/Fe_3_O_4_ shows an increase in current density up to 0.6 M ethanol (Fig. 7c). This increasing trend is observed up to 0.8 M ethanol in the NiO/Fe_3_O_4_/rGO modified electrode in an alkaline medium (Fig. 7d). Beyond these concentrations, a decrease in current density is observed in the EOR, indicating catalyst surface saturation beyond a critical concentration, leading to a declining trend in current density.

**Fig. 8:**
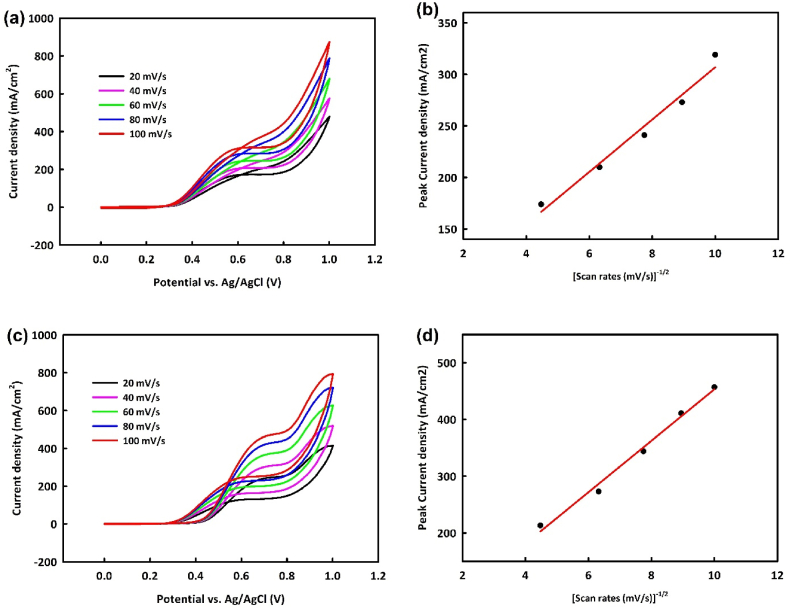
Cyclic voltammetry analysis in the potential range of 0–1 V at the different scan rates in optimal concentrations of Methanol/1M KOH for NiO/Fe_3_O_4_ (a) and NiO/Fe_3_O_4_/rGO (c) and the square root of scan rates against the oxidation peak current density for NiO/Fe_3_O_4_ (b) and NiO/Fe_3_O_4_/rGO (d).

With the optimal concentration of 0.6 M methanol for NiO/Fe_3_O_4_ (NF) and 0.8 M methanol for NiO/Fe_3_O_4_/rGO (NFR) in a 1 M KOH solution, the behavior of these two catalysts will be examined at various scan rates. Fig. 8a and c shows an increase in faradaic and capacitive currents with increasing scan rates from 20 to 100 mV/s for NiO/Fe_3_O_4_ and NiO/Fe_3_O_4_/rGO, respectively. Plotting the square root of scan rates against the oxidation peak current density (Fig. 8b and d) for both catalysts indicates a linear relationship with R^2^ = 0.995 and R^2^ = 0.998, suggesting a diffusion-controlled mechanism for these catalysts in the MOR. The envisioned mechanism for methanol oxidation is as follows:

$$NFR/NF+MeOH\to NFR/NF-{MeOH}_{ads}$$

$$NFR/NF-{MeOH}_{ads}+4OH^{-}\to NFR/NF-{\left(CO\right)}_{ads}+4H_{2}O+4e^{-}$$

$$NFR/NF+OH^{-}\to NFR/NF-{OH}_{ads}+e^{-}$$

$$\text{NFR}/\text{NF}-{CO}_{ads}+\text{NFR}/\text{NF}-{OH}_{ads\;}+OH^{-}\;\to \text{NFR}/\text{NF}+{CO}_{2}+H_{2}O+e^{-}$$

**Fig. 9:**
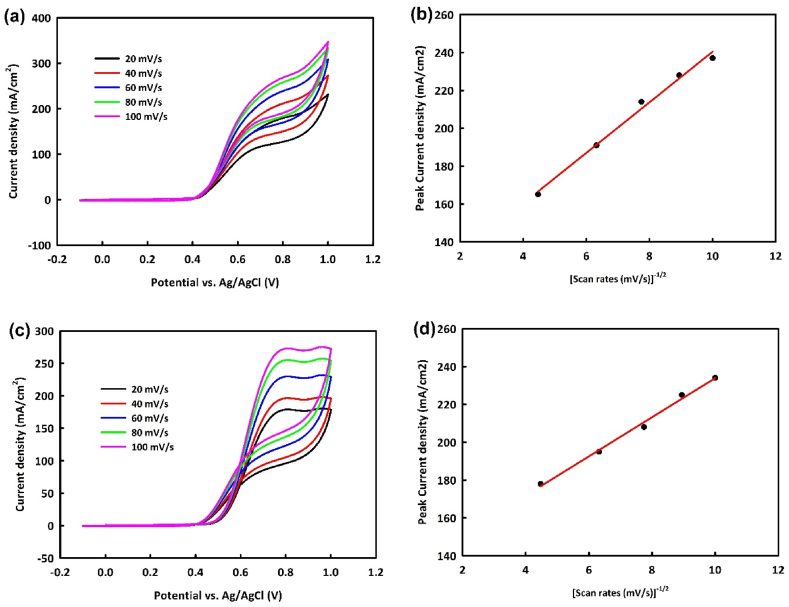
Cyclic voltammetry analysis in the potential range of 0–1 V at the different scan rates in optimal concentrations of ethanol/1 M KOH for NiO/Fe_3_O_4_ (a) and NiO/Fe_3_O_4_/rGO (c) and the square root of scan rates against the oxidation peak current density for NiO/Fe_3_O_4_ (b) and NiO/Fe_3_O_4_/rGO (d).

The EOR mechanism was also examined by performing CV analysis with NiO/Fe_3_O_4_ and NiO/Fe_3_O_4_/rGO at various scan rates. In ethanol oxidation, an increasing trend in oxidation current density with increasing scan rates is observed for both catalysts (Fig. 9a and c). A proportional relationship between the oxidation peak current density and the square root of scan rate with R^2^ = 0.992 and R^2^ = 0.996 is also noted (Fig. 9b and d), indicating a diffusion-controlled mechanism. The proposed ethanol oxidation mechanism is as follows:

$$\text{NFR}/\text{NF}+OH^{-}\to \text{NFR}/\text{NF}-{\text{OH}}_{ads}+e^{-}$$

$$\text{NFR}/\text{NF}+\text{EtOH}\to \text{NFR}/\text{NF}-{\left(\text{EtOH}\right)}_{ads}$$

$$\text{NFR}/\text{NF}-{\left(\text{EtOH}\right)}_{ads}+3{\text{OH}}^{-}\to \text{NFR}/\text{NF}-{\left({\text{CH}}_{3}\text{CO}\right)}_{ads\;}{+3H}_{2}O+3e^{-}$$

$$\text{NFR}/\text{NF}-{\left({\text{CH}}_{3}CO\right)}_{ads}+\text{NFR}/\text{NF}-{\text{OH}}_{ads}\to \text{NFR}/\text{NF}-{\left({\text{CH}}_{3}\text{COOH}\right)}_{ads\;}+\text{NFR}/\text{NF}$$

$$\text{NFR}/\text{NF}-{\left({\text{CH}}_{3}COOH\right)}_{ads}+OH^{-}\to \text{NFR}/\text{NF}+{\text{CH}}_{3}\text{COO}‾{+H}_{2}O$$

In explaining the MOR and EOR mechanisms, it is noted that in the first stage, methanol/ethanol is adsorbed onto the catalyst surface and in its pores. In the next stage, the adsorbed methanol/ethanol undergoes deprotonation in the presence of hydroxyl ions, producing by-products such as (CH₃O, CH₂O, CHO) and (CH₃CH₂O, CH₃CHO, CH₃CO). Additionally, hydroxyl ions are adsorbed onto the catalyst surface. In the final stage, the catalyst surface is cleared of the adsorbed species, allowing the catalyst to re-engage in the oxidation process.

**Fig. 10:**
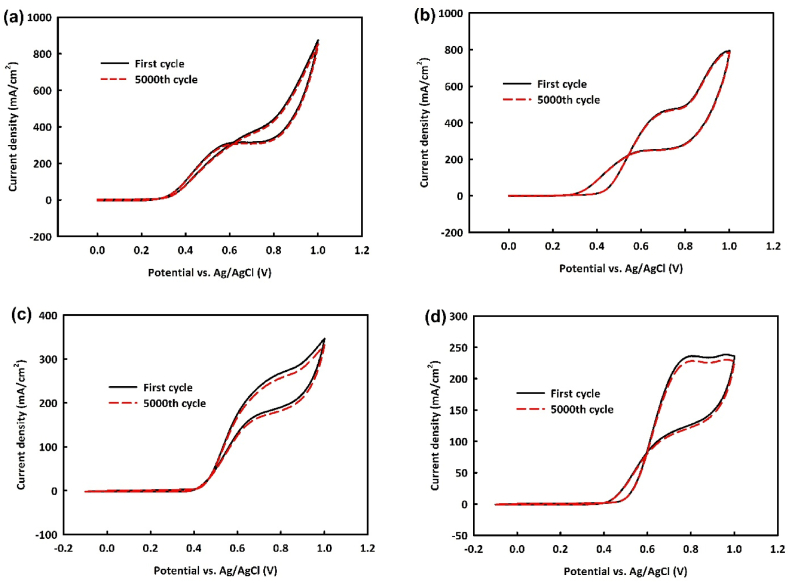
Cyclic stability of NiO/Fe_3_O_4_ and NiO/Fe_3_O_4_/rGO catalysts in the MOR (a and b) and in EOR (c and d) in the optimal concentration of alcohol in the scan rate of 100 mV/s.

The stability of NiO/Fe_3_O_4_ and NiO/Fe_3_O_4_/rGO catalysts in the MOR was examined by performing 5000 consecutive CV cycles at optimal methanol concentration and a scan rate of 100 mV/s. As seen in Fig. 10a and b, the nanocatalysts exhibit very good stability of 97.3 % and 98.9 % respectively, after this many consecutive cycles have been run. In the EOR, Fig. 10c and d shows cycle stability of 95.8 % and 96.6 %, respectively. Overall, both catalysts demonstrate very good stability in alcohol oxidation, with the rGO-containing catalyst showing slightly better stability. This improved stability could be attributed to the increased active surface area provided by rGO, which allows the catalyst to offer more active sites to the electrolyte and fuel, delaying saturation by alcohol oxidation by-products.

**Fig. 11:**
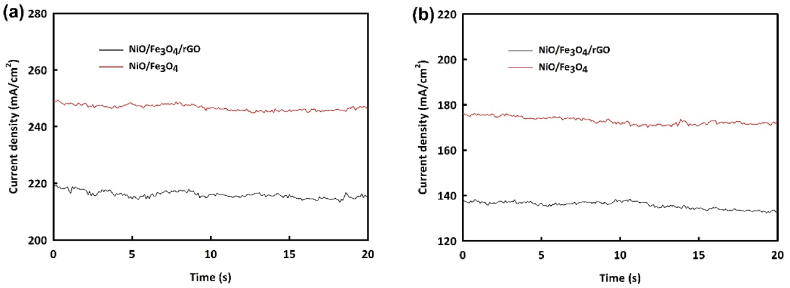
Chronoamperometry analysis over 20 h for NiO/Fe_3_O_4_ and NiO/Fe_3_O_4_/rGO catalysts in the MOR (a) and EOR (b).

Chronoamperometry analysis over 20 h was performed to further investigate the stability of synthesized catalysts at optimal methanol and ethanol concentrations and peak potentials. As seen in Fig. 11a, NiO/Fe_3_O_4_ shows a stability of 97.5 % and NiO/Fe_3_O_4_/rGO shows 98.7 % in methanol oxidation. In ethanol oxidation, they show 95.35 % and 96.4 % stability, respectively, indicating excellent values (Fig. 11b).

**Fig. 12:**
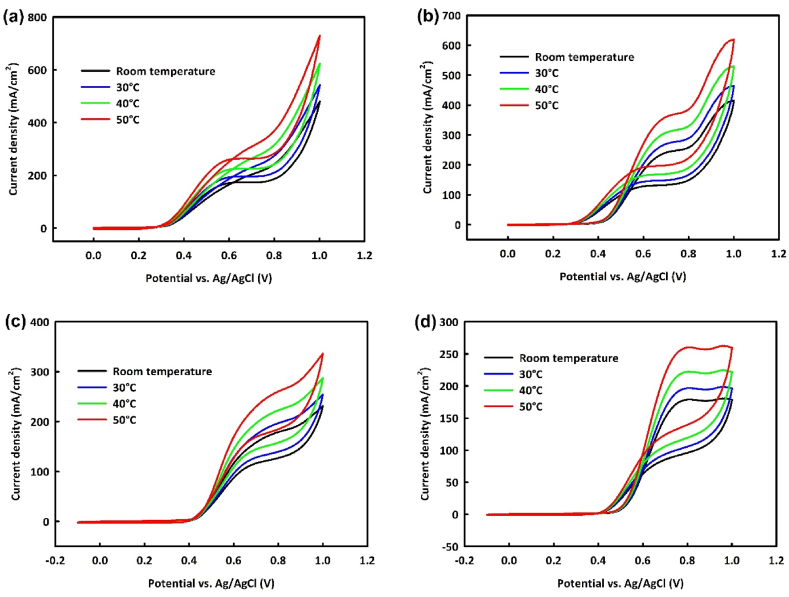
Cyclic voltammetry analysis at different temperatures ranging from room temperature to 50 °C in the scan rate of 20 mV/s for NiO/Fe_3_O_4_ and NiO/Fe_3_O_4_/rGO in the MOR (a and b) and EOR (c and d).

One advantage of alcohol fuel cells is their good performance at low temperatures. To examine the behavior of NiO/Fe_3_O_4_ and NiO/Fe_3_O_4_/rGO catalysts in MOR and EOR at various temperatures, CV analysis was performed on modified electrodes at different temperatures ranging from room temperature to 50 °C in the scan rate of 20 mV/s. Fig. 12a shows CV analysis of NiO/Fe_3_O_4_ at optimal methanol concentration with increasing temperature, indicating an increase in oxidation current density. This trend is also observed for NiO/Fe_3_O_4_/rGO within the same temperature range (Fig. 12b). In EOR (Fig. 12c and d), increasing current density with temperature is observed for both catalysts at optimal ethanol concentration. It appears that with increasing temperature, methanol and ethanol more readily release their OH groups, facilitating alcohol oxidation processes overall.

## 4 Conclusion

The superior electrical conductivity and surface activity of reduced graphene oxide (rGO) prompted its consideration as a support for two metal catalysts in this study. The ternary catalyst comprising nickel oxide, iron oxide, and rGO was synthesized via a one-step hydrothermal method and evaluated as a supercapacitor electrode and anode catalyst in methanol and ethanol fuel cells. rGO improved the performance of nanocomposites in both energy storage and generation applications. The electrode materials NiO/Fe_3_O_4_/rGO and NiO/Fe_3_O_4_ showed specific capacitances of 946 F/g and 1155 F/g at the current density of 1 A/g and cyclic stability of 83.6 % and 90.6 %, after 10000 GCD cycles, making them stable and cost-effective electrodes for energy storage. Both nanocomposites also demonstrated excellent performance in methanol and ethanol oxidation, with NiO/Fe_3_O_4_/rGO showing a current density of 450 mA/cm^2^ and stability of 98.7 % in chronoamperometry over 20 h in methanol oxidation. NiO/Fe_3_O_4_/rGO in ethanol oxidation showed an oxidation current density of 235 mA/cm^2^ and stability of 96.4 %, making it a serious candidate for use in alcohol fuel cell anodes. NiO/Fe_3_O_4_/rGO and NiO/Fe_3_O_4_ composites demonstrated excellent energy storage and generation potential and can be considered for industrial applications.

## CRediT authorship contribution statement

**Mohammad Bagher Askari:** Writing – original draft, Supervision, Investigation, Formal analysis, Data curation. **Mohammad Taghi Tourchi Moghadam:** Writing – review & editing, Software, Project administration, Data curation. **Parisa Salarizadeh:** Writing – review & editing, Validation, Investigation, Formal analysis.

## Data availability statement

Data are available on request from the corresponding author (MB. Askari).

## Declaration of competing interest

The authors declare that they have no known competing financial interests or personal relationships that could have appeared to influence the work reported in this paper.
